# GHG Monitoring Project for the Global Stocktake 2023: implications of the COP26 Japan Pavilion seminar

**DOI:** 10.1186/s13021-022-00211-z

**Published:** 2022-07-18

**Authors:** Tomohiro Oda

**Affiliations:** 1 Earth From Space Institute, Universities Space Research Association, Columbia, MD US; 2 Department of Atmospheric and Oceanic Science, University of Maryland, College Park, MD US; 3 Graduate School of Engineering, Osaka University, Suita, Osaka Japan

**Keywords:** COP26, Global Stocktake, Greenhouse gas (GHG), Climate mitigation, Emission monitoring

## Abstract

During the 2021 Glasgow Climate Change Conference (COP26), a hybrid seminar event “Greenhouse gas (GHG) Monitoring Project for the Global Stocktake 2023” was held at the COP26 Japan Pavilion on 2nd of November 2011. The participants presented and discussed science-based GHG monitoring approaches in support of the Global Stocktake. This report summarizes the five research talks given at the event.

## Background

**Fig. 1:**
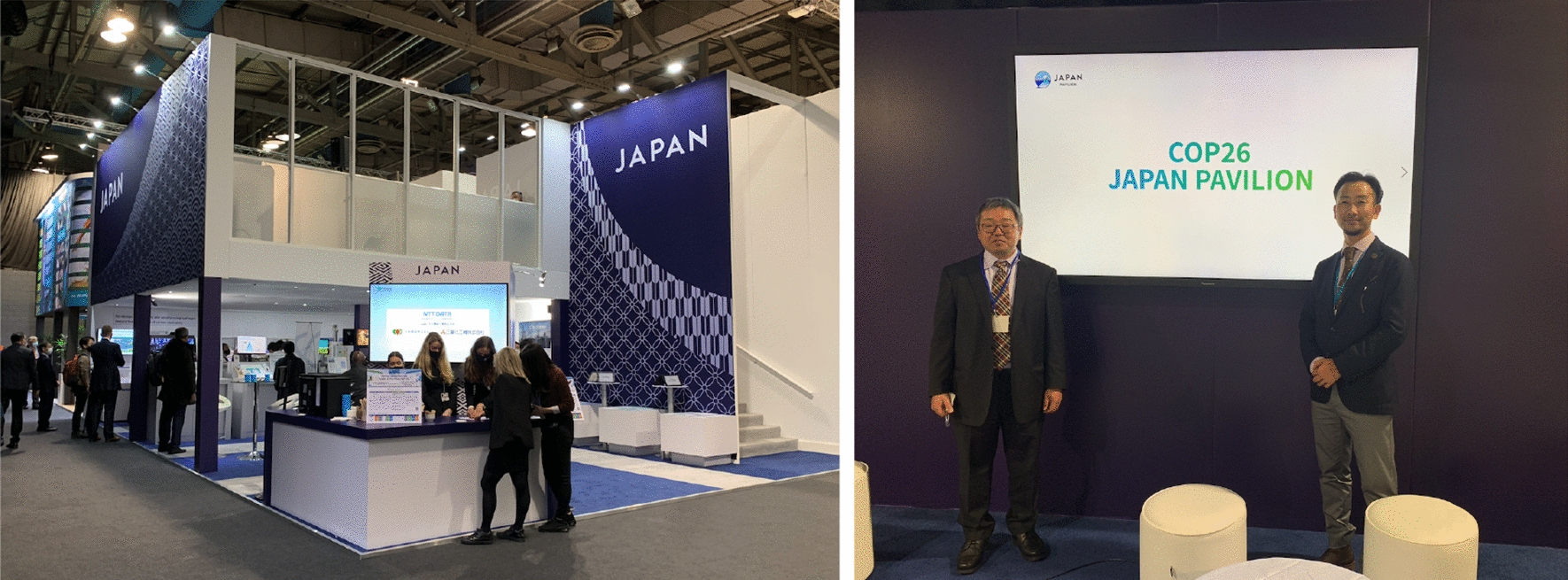
The COP26 Japan Pavilion (left) and the seminar venue on the second floor (right, with Akihiko Ito)

The 26th session of the Conference of the Parties (COP 26) to the United Nations Framework Convention on Climate Change (UNFCCC) was held in Glasgow, UK. The Ministry of the Environment Japan (MOEJ) hosted a hybrid (live and virtual) event titled “GHG Monitoring Project for the Global Stocktake 2023” on 2nd of November with the Japan Aerospace Exploration Agency (JAXA) and the National Institute for Environmental Studies (NIES), which served as co-hosts (see Fig. 1).

The Global Stocktake (GST, [1]) is a process for assessing the progress towards the implementation of the Paris Agreement and updating its long-term goals for reducing total greenhouse gas (GHG) emissions to mitigate increases in global temperature. GSTs are to be conducted at five-year intervals, starting in 2023. The Parties to the Paris Agreement have not yet reached a consensus on its exact procedure for conducting the first GST, but they have agreed that it should be based on the best available science and conducted in the context of an enhanced transparency framework.

At the MOEJ event, we showcased and discussed actionable items, and discussed the knowledge and technological gaps that must be addressed between now and the GST. The event consisted of five science talks and a panel discussion (see Table 1). This report briefly summarizes the five talks. Further information on the MOEJ event at the COP26 is available from a NIES webpage [2].

**Table 1:** Agenda of the seminar event

	Speaker	Presentation title
Part 1: Scientific highlights and knowledge gaps	Akihiko Ito (NIES)	Project for the Global Stocktake 2023
	Hiroshi Suto (JAXA)	Decade-long global GHG observations by GOSAT towards the Global Stocktake
	Parbir Patra (JAMSTEC)	Satellite data helping estimation and evaluation of regional CO_2_ and CH_4_ fluxes
	David Crisp (JPL/Caltech)	Pilot national-scale estimates of carbon dioxide and methane emissions and removals from space-based measurements
	Masataka Watanabe (Chuo University)	The UNFCCC Biennial Update Report (BUR) preparation in Mongolia using GOSAT satellite data and its application to other countries
Part 2: Panel discussion	*Q1. How can we utilize the knowledge and information you provide form your research to support the current emission reporting and assess our climate mitigation progress towards GST2023?*	
	*Q2. What changes in society would like to see/think need to happen/should we see, and how do you detect them and/or how do we make those changes happen?*	
	Panelists: Ito, Suto, Patra, Crisp, Watanabe, Nobuko Saigusa (NIES), Moeko Yoshitomi (MOEJ); Moderator: Tomohiro Oda (USRA)	

## Main text

Akihiko Ito (NIES) presented an overview of a newly launched research activity by the Japanese research community with a focus on an MOEJ-funded project titled “Comprehensive Study on Multi-scale Monitoring and Modeling of Greenhouse Gas Budget.” With a clear intention to support the first GST in 2023, the project is composed of three themes: (I) atmospheric measurement and top-down estimation of GHG budgets, (II) evaluation and mitigation assessments with an Earth system model, and (III) bottom-up estimation of GHG budget using inventories and biogeochemical models. The project is intended to extensively cover the Asia-Pacific region by ground, ship, aircraft measurements, leveraging the observational networks maintained by the NIES and the Meteorological Research Institute (MRI), Japan. In addition, the long-term space-based GHG data collected by the Greenhouse gas Observing SATellite (GOSAT) project satellites are being used to elucidate regional emissions and removals of CO_2_ and CH_4_ globally. Ito also highlighted advanced models are being used in both top-down and bottom-up studies: the Nonhydrostatic ICosahedral Atmospheric Model (NICAM) and the Model for Interdisciplinary Research on Climate Earth System Model (MIROC) for atmospheric data inversion, and the Vegetation Integrative Simulator for Trace gases (VISIT) and VISIT coupled in the MIROC Earth system model for land ecosystem process-based exchanges of GHGs. By integrating observations and modeling, the project plans to show a comprehensive decadal and spatial analysis of global and regional/national GHG budgets. The project also aims to provide a timely multi-scale, sector-specific reporting of GHG emissions and removals in order to contribute to the first GST and consecutive events. In addition to a summary report that will be published for policymakers, the research outcome (observational data and analysis/modeling results) from this project will be made available on open data repositories for public use.

Hiroshi Suto (JAXA) presented Japan’s decade-long space-based observations by GOSAT, the first dedicated satellite for measuring GHG sources and sinks on regional scales. Since its launch in January 2009, GOSAT has continuously collected CO_2_ and CH_4_ data globally every 3 days using both sunlight reflected from the Earth’s surface in the shortwave infrared (SWIR) bands and thermal emission in the thermal infrared (TIR) bands. With the launch of GOSAT-2 in October 2018, both satellites are continuing measurements of CO_2_ and CH_4_ along with other complementary variables. GOSAT has an agile, 2-axis pointing system that allows it to target intense emission sources due to human activities, such as power plants and more than 50 megacities around the world. Recently, by utilizing both reflected sunlight and thermal emissions observed by GOSAT, the JAXA Earth observation Research Center has developed an algorithm to derive partial-column information with an aim to better support surface emission estimates. Suto showed the CO_2_ changes over Tokyo and Beijing for January through April of each year from 2016 through 2020 derived from these new products. The results show that all months in 2020 have reduced CO_2_ enhancements relative to prior years, a behavior that is consistent with the reported reductions of fossil fuel emissions associated with the COVID-19 lockdowns. This suggests that the new products have a potential to capture the anthropogenic emissions over megacities more clearly than conventional total-column approaches. Currently, JAXA is reprocessing the GOSAT data using the new retrieval algorithm and will deliver the upper and lower partial columns, and total column products to the research community, which will accelerate our understanding of the carbon cycle toward GST.

Prabir Patra (Japan Agency for Marine-Earth Science and Technology, JAMSTEC) discussed the top-down methods for quantifying GHG fluxes. As done under the Kyoto Protocol since 1997, parties to the Paris Agreement need to compile and report national inventories of GHG emissions and removals to the UNFCCC. These inventories use bottom-up methods to estimate annual net GHG emissions and removals from specific sectors specified in the Intergovernmental Panel on Climate Change (IPCC) Guidelines. GHG emissions and removals can also be estimated from spatially- and temporally-resolved measurements of their atmospheric concentrations using inverse modeling systems (top-down method). The top-down inverse modeling analyses using the atmospheric chemistry transport model (ACTM) have been significantly advanced over the past 3 decades. Patra has worked on top-down estimations of GHG (CO_2_, CH_4_ and N_2_O) using long-term observations from surface sites using MIROC-ACTM. This approach corrects trends in regional emissions and removals derived from bottom-up estimates of anthropogenic emissions and natural exchanges between the land/ocean surfaces and atmosphere. Patra’s results strongly suggest that a joint use of bottom-up GHG inventories with the top-down estimates of net GHG emissions would provide a more accurate and complete assessment of the regional ‘net fluxes’ (emissions–removals), as well as greater insight into the underlying processes. If estimates reported by bottom-up inventories have systematic biases, these biases can be detected and then corrected using the top-down inversion modeling results. As an existing challenge, Patra noted that uncertainties in the top-down net flux estimation for CH_4_ and N_2_O arise from atmospheric model transport errors and calculation of photo-chemical loss in the troposphere and the stratosphere. He also noted that additional checks on model transport, hydroxyl (OH) radical abundance for main CH_4_ loss, and photolysis in the stratosphere for N_2_O must be performed and validated using independent tracers in order to ensure transparency of the reported emission and removal values.

David Crisp (Jet Propulsion Laboratory/California Institute of Technology, JPL/Caltech) discussed the ongoing effort by the Committee for Earth Observation Satellites (CEOS) Atmospheric Composition Virtual Constellation (AC–VC) and the Joint CEOS/CGMS WGClimate Greenhouse Gas Task Team to develop pilot, top-down inventory products to support the 2023 GST [3]. Following Patra, Crisp further noted that the top-down CO_2_ and CH_4_ budgets, or “atmospheric inventories,” are not as process-specific as bottom-up inventories, but complement them by providing an integrated constraint on net emissions and removals from all processes across a wide range of spatial scales. They also track emission changes in the natural biosphere and ocean due to human activities and climate change. Recent progress in ground-based, airborne and space-based CO_2_ and CH_4_ measurement and modeling capabilities have substantially enhanced the value of top-down methods for both inventory development and assessment applications. To demonstrate these advances, CO_2_ obtained from the NASA Orbiting Carbon Observatory-2 (OCO-2) are being combined with ground-based and airborne CO_2_ measurements. The OCO-2 Science Team is analyzing these data with ensemble of state-of-the-art CO_2_ flux inversion systems that use different transport models, meteorology, and inverse methods to yield national-scale estimates of net CO_2_ emissions. In parallel, the NASA Carbon Monitoring System (CMS) Flux Team has derived the national-scale CH_4_ budgets by analyzing remote sensing observations from GOSAT. The primary objective of this activity is to start a conversation with stakeholders and users to establish the utility and best practices for combining bottom-up and top-down methods to enable a more complete and transparent GST.

Masataka Watanabe (Chuo University, Japan) further discussed the use of the top-down estimates in support of countries’ UNFCCC reporting by using his research project in Mongolia as an example. While the compilation and reporting of Biennial Update Reports (BURs) and National Inventory Reports (NIRs) are an essential requirement under the Paris Agreement, a significant portion of Non Annex I countries did not yet submit them to the UNFCCC, which is largely due to knowledge and resource gaps, such as collection of field data in frequent intervals and upscaling to country totals. In support of the emission report compilation, the use of satellite-based top-down inverse estimates of GHG emissions and removals has been examined. Japan’s Chuo University and Mongolia’s Information and Research Institute for Meteorology, Hydrology, and Environment jointly developed an inverse modeling system that is based on the Weather Research and Forecasting (WRF) regional transport model and the Vegetation Photosynthesis Respiration Model (VPRM). The system is designed for top-down estimation of CO_2_ emissions and removals using data collected by GOSAT satellites. The system domain is set to Mongolia as a whole, accommodating the city of Ulaanbaatar (the capital of Mongolia), where CO_2_ is mainly being emitted from coal-fired power plants and heating boilers, which account for about 75% of Mongolia’s national total CO_2_ emissions. Watanabe’s top-down estimate suggests a potential bias in the annual total CO_2_ emissions reported for 2018. The top-down information has been considered to be used for the next compilation of Mongolia's BUR. Watanabe concluded that the method for satellite-based estimating GHG inventories co-developed with Mongolia is a good example for demonstrating the science-based approaches in support of the UNFCCC implementation. In theory, the method could be applicable to other countries who also share challenges due to knowledge and resource gaps.

## Conclusions

Towards the upcoming GST 2023, there are ongoing research efforts to integrate observations and modeling in order to establish systems to monitor the GHG budget. Actionable information/products have started to become available from such GHG monitoring systems in development. The information/products are being actively examined for the purpose of monitoring our climate mitigation progress and informing decision making. This event identified and discussed the existing technological and knowledge gaps through global collaboration towards robust GHG monitoring in support of GST. In addition, this event also highlighted the importance of filling the resource gaps among countries and regions in order to guide our global climate mitigation effort towards the Paris Agreement goal.

## Data Availability

Further information on the Japan Pavilion seminar event at the COP26 is available at (https://esd.nies.go.jp/cop26/seminar-2-11-2021.html).
